# Sintilimab-related diabetes mellitus and psoriasis: A case report and literature review

**DOI:** 10.1097/MD.0000000000035946

**Published:** 2023-11-10

**Authors:** Wenying Huang, Yan Liu, Min Li, Yuan Xue, Weichao Bao, Ying Guo

**Affiliations:** a School of Clinical Medicine, Weifang Medical University, Affiliated Hospital of Weifang Medical University, Weifang, Shandong Province, China; b Department of Endocrinology and Metabolic Diseases, Affiliated Hospital of Weifang Medical University, Weifang, Shandong Province, China.

**Keywords:** diabetes mellitus, immune-related adverse events, lactic acidosis, psoriasis, Sintilimab

## Abstract

**Rationale:**

With the popularity of ICIs in different oncology treatments, immune-related adverse events have raised concerns, mostly occurring in skin and endocrine gland injury. This disease involves different organ systems and presents with a variety of clinical manifestations. Most patients with immune checkpoint inhibitor-induced type 1 diabetes are reported to have no combination of autoimmune disease. We report a case of Sintilimab-related diabetes mellitus and psoriasis.

**Patient concerns:**

We report a case of a 65-year-old female with Sintilimab-related diabetes mellitus and psoriasis.

**Diagnosis:**

The patient treated with anti-programmed cell death protein 1 (Sintilimab) for 4 cycles. The patient presented with inexplicable bouts of nausea and vomiting, accompanied by chest discomfort and a feeling of breathlessness, prompting their admission to the local hospital. The initial assessment upon admission revealed an abrupt elevation in blood glucose levels, alongside normal ketone levels, lactic acidosis, and hyperuricemia. A comprehensive regimen was provided to regulate glucose levels and address the symptoms, resulting in notable improvement and subsequent discharge. Regrettably, the patient’s personal decision to discontinue medication for a single day led to the emergence of acute ketoacidosis, coupled with a recurrence of psoriasis vulgaris. Consequently, readmission became necessary. Based on the patient’s medical history and diabetes antibody testing, the diagnosis of immune checkpoint inhibitor induced diabetes mellitus has been confidently established.

**Interventions:**

The patient ceased treatment with Sintilimab and was initiated on insulin therapy for glycemic control, alongside symptomatic management for psoriasis. Upon stabilization of the condition, long-term administration of exogenous insulin was implemented as a substitute treatment.

**Outcome:**

Outside of the hospital, insulin therapy effectively maintained stable blood glucose levels, and there were no further episodes of psoriasis flare-ups.

**Lesson:**

The clinical manifestations of immune checkpoint inhibitor induced diabetes mellitus are variable, and in this case the patient presented with unique primary symptoms. Therefore, it is crucial to accumulate relevant cases, understand the different clinical presentations and identify the underlying mechanisms of the disease. This will provide further evidence for early therapeutic intervention in similar patients in the future.

## 1. Introduction

Immune checkpoints play a critical role in suppressing the activation of T cells, thereby limiting their ability to recognize and eliminate tumor-associated antigens. By exploiting immune checkpoints, tumor cells can evade immune surveillance and clearance. Immune checkpoint inhibitors (ICIs) work by blocking these inhibitory pathways, effectively “releasing the brakes” on the immune system and reinvigorating its antitumor response. However, ICIs can also lead to immune-related adverse events (irAEs), where the immune system attacks not only the tumor but also healthy tissue. The skin and endocrine glands are commonly affected organs, with skin reactions often manifesting as pruritus and rash, although reported cases of psoriasis are rare.^[1]^ ICIs can also affect the pancreas, resulting in ICIs induced diabetes mellitus (ICI-DM), which occurs in <1% of cases^[2,3]^ and presents with a variety of initial symptoms. In cases where the diagnosis of ICI-DM is not immediately apparent, it can delay appropriate treatment and pose potential risks to the patient’s life.^[4]^ This report describes a case in which the administration of a programmed cell death protein 1 (PD-1) inhibitor (Sintilimab) to a patient with squamous cell lung cancer triggered a flare-up of psoriasis vulgaris and the development of new-onset diabetes mellitus. In contrast to the typical symptoms of diabetic ketoacidosis, this patient with ICI-DM presented with hyperglycemia, hyperuricemia, and lactic acidosis. Because patients with a history of autoimmune disease are generally excluded from ICI clinical trials, there are limited reported cases of concurrent multiple irAEs that should be carefully considered by clinicians.

## 2. Case presentation

The patient, a 65-year-old female, presented with a sudden onset of symptoms of nausea and vomiting over the past 3 days. She experienced difficulty in vomiting undigested food, as well as chest tightness and shortness of breath. Notably, she did not have the typical symptoms of diabetes such as excessive thirst (polydipsia), frequent urination (polyuria) or increased appetite (polyphagia). Furthermore, she had lost approximately 5 kg of weight over the past month. The patient had a background of “right lung squamous cell carcinoma” for over 4 months, which had been treated with paclitaxel + cisplatin + Sintilimab for 4 cycles. She also had a long-standing history of psoriasis, spanning more than 40 years, during which she did not experience any flare-ups. There was no known history of diabetes mellitus. Her menstrual and marital histories were unremarkable. The patient had a history of smoking, having quit 40 years ago, with a total exposure of 365 pack-years. There was no reported family history of diabetes or related genetic disorders in her first- or second-degree relatives. On physical examination, her pulse rate was recorded as 138 beats per minute, blood pressure was 97/66 mm Hg, respiratory rate was 22 breaths per minute, and oxygen saturation was 92% on low-flow oxygen therapy. Coarse breath sounds were auscultated in both lungs, and the extremities exhibited scaly erythema, papules, wax drip phenomenon (+), film phenomenon (+), and punctate hemorrhage phenomenon (+) of varying sizes. The remainder of the physical examination was unremarkable.

Laboratory tests revealed several abnormalities. In particular, blood ketones, thyroid hormones, sex hormones, adrenocorticotropic hormones, and cardiac enzyme profiles were within normal ranges. However, the patient’s blood glucose level was markedly elevated at 43.11 mmol/L (normal range: 3.8–6.1 mmol/L), indicating hyperglycemia. The glycosylated hemoglobin level was also elevated at 8.1% (normal range: 4.2–6.2%), indicating long-term elevated blood glucose levels. Serum C-peptide levels were reduced to 0.34 ng/mL (normal range: 1.1–4.4 ng/mL), indicating impaired insulin production. Furthermore, the uric acid level was increased to 608.0 μmol/L (normal range: 135–425 μmol/L), the blood sodium level was decreased to 121.2 mmol/L (normal range: 137–147 mmol/L), the blood potassium level was increased to 5.9 mmol/L (normal range: 3.5–5.3 mmol/L), and the anionic gap level was increased to 39.8 mmol/L (normal range: 8–16 mmol/L). Analysis of urine samples showed the presence of ketones (3+), sugar (3+), and protein (+). Arterial blood gas analysis showed an abnormal pH of 7.04 (normal range: 7.38–7.42), a decreased partial pressure of carbon dioxide of 14 mm Hg (normal range: 38–42 mm Hg) and an elevated lactate level of 2.8 mmol/L (normal range: 0.5–1.6 mmol/L). Furthermore, the patient tested negative for anti-islet cell antibodies, glutamic acid decarboxylase antibodies, insulin autoantibodies, tyrosine phosphatase antibodies, and zinc transporter 8 antibodies.

**Figure 1.**
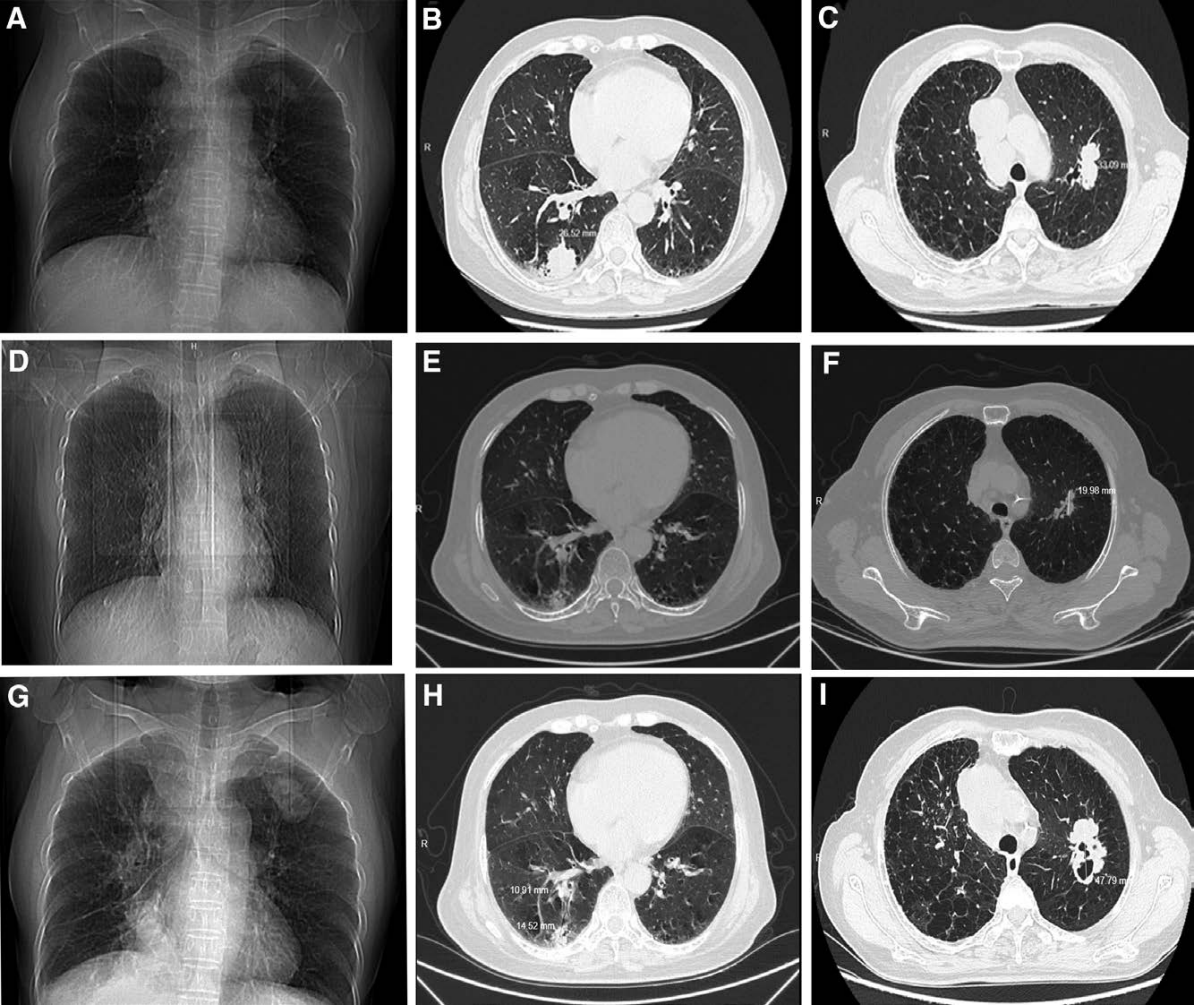
Chest computed tomography (CT) scans of a 65-year-old female patient before and after treatment with ICIs. (A–C) Pretreatment chest CT (August 30, 2021); (D–F) Posttreatment chest CT (December 06, 2021); (G–I) Chest CT after 9 months of treatment interruption (September 14, 2022).

**Figure 2.**
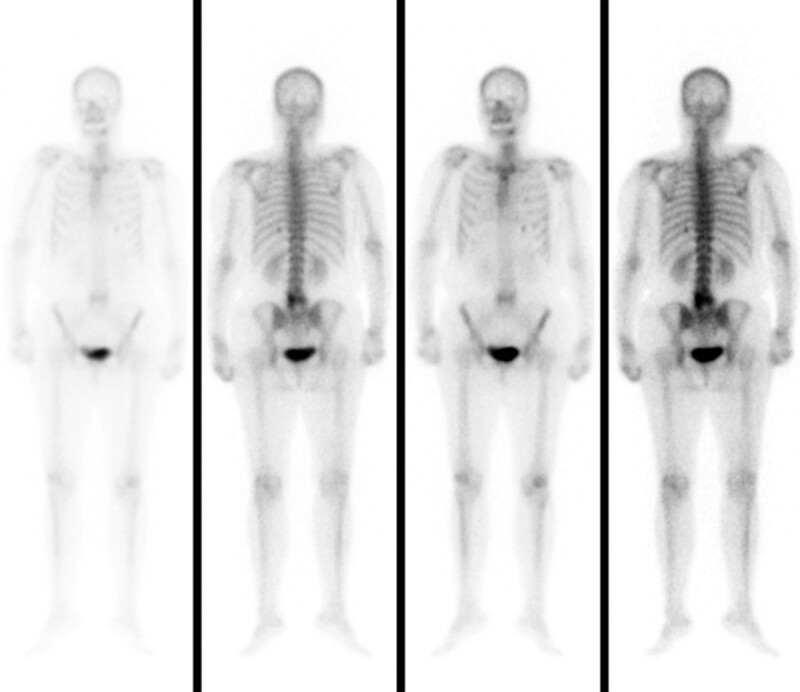
Whole-body bone imaging showed radiological elevation of the left arch of L5, suggesting the presence of potential bone metastases.

An enhanced CT scan of the chest (Fig. 1A–I) showed bronchiectasis involving both lungs and lung cancer with metastases to the right hilar and mediastinal lymph nodes. Magnetic resonance imaging of the abdomen showed possible scattered lymph node metastases in the retroperitoneal, pelvic, and bilateral inguinal regions. Whole-body bone imaging (Fig. 2) showed radiological elevation of the left arch of L5, suggesting possible bone metastases. The patient was diagnosed with immune checkpoint inhibitor induced type 1 diabetes, lactic acidosis, hyperuricemia, and psoriasis vulgaris. The treatment approach included insulin administration to regulate glucose levels, rehydration to correct electrolyte imbalances, and symptomatic treatment of psoriasis with pavoline, anti-silver granules, and narrow-spectrum ultraviolet radiation. However, 2 days after discontinuing insulin without medical advice, the patient was readmitted for diabetic ketoacidosis and Sintilimab immunotherapy was discontinued. On discharge, improvements in the patient’s scaly erythema and papular rash on the extremities were noted, while the remaining physical examination findings were largely unchanged. Laboratory tests revealed a blood glucose level of 5.70 mmol/L (normal range: 3.8–6.1 mmol/L), a blood ketone level of 0.13 mmol/L (normal range: 0.03–0.3 mmol/L), a serum C-peptide level of <0.01 ng/mL (normal range: 1.1–4.4 ng/mL), a serum insulin level of <0.20 μmol/L (normal range: 3.21–16.32 μmol/L), uric acid level of 301.0 μmol/L (normal range: 135–425 μmol/L), blood sodium level of 140.2 mmol/L (normal range: 137–147 mmol/L), and blood potassium level of 4.7 mmol/L (normal range: 3.5–5.3 mmol/L). Arterial blood gas analysis revealed a pH of 7.35 (normal range: 7.38–7.42) and a lactate level of 1.2 mmol/L (normal range: 0.5–1.6 mmol/L). The patient continued subcutaneous insulin to manage glycemia and received oral anti-silver granules to control psoriasis progression at home.

Nine months later, the patient’s glucose-lowering regimen remained unchanged, the psoriasis medication had been discontinued, and all laboratory results were within normal ranges except for a serum C-peptide level of <0.01 ng/mL (normal range: 1.1–4.4 ng/mL) and a serum insulin level of <0.20 μmol/L (normal range: 3.21–16.32 μmol/L). The patient had been discontinuing Sintilimab treatment for approximately 1 year and was effectively managing glycemia with insulin therapy. Additionally, there were no indications of psoriasis recurrence. Unfortunately, the patient passed away in December 2022 due to advanced pulmonary cancer and associated complications.

## 3. Discussion

**Figure 3.**
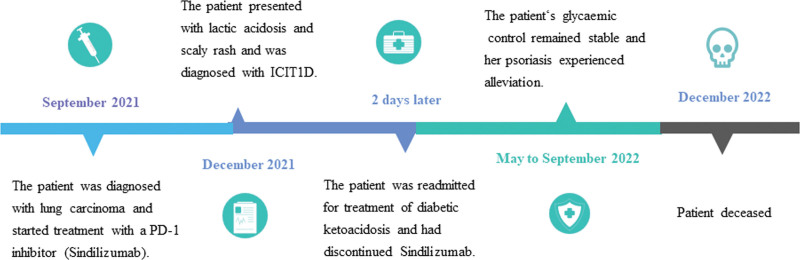
Disease progression of disease timeline.

In 2015, Martin-Liberal et al^[5]^ first reported a case of autoimmune diabetes mellitus in a melanoma patient following administration of a PD-1 inhibitor. Since then, further reports of ICI-DM have emerged.^[6,7]^ Clinical trials involving ICIs typically exclude individuals with preexisting autoimmune diseases, and most irAEs are usually documented as new adverse events. In this particular case, the patient not only developed new-onset diabetes mellitus after receiving Sintilimab, but also experienced worsening psoriasis symptoms. Just 2 days after stopping insulin therapy alone (Fig. 3), the patient was readmitted to hospital with diabetic ketoacidosis. This acute and transient islet dysfunction is consistent with the characteristics of fulminant type 1 diabetes. Taking into account the patient’s psoriasis and the administration of the PD-1 inhibitor Sintilimab, the following pathogenesis can be proposed.

The investigation into the mechanisms of tumor immune evasion has led to significant interest in the treatment of tumors and irAEs through ICIs.^[8]^ Immune checkpoints can be likened to “balance points” within the immune system. They prevent the decline of immune function, which could otherwise create opportunities for pathogens to invade the body. Additionally, they inhibit excessive immune responses that may lead the immune system to attack its own tissues. Among the known immune checkpoints, PD-1 is primarily expressed on activated T cells, although it can also be found on hematopoietic cells, vascular endothelial cells, and islet cells. By binding to programmed death-ligand l (PD-L1), PD-1 can effectively restrain pathological autoimmune tissue damage and suppress the release of cytokines.^[9]^ In essence, the PD-1/PD-L1 pathway acts as a “brake” in the immune response, and the use of ICIs functions as a means of “releasing the brake.” PD-L1 expressed on human islet cells safeguards them from attacks by immune cells, while tumors exploit this system by expressing the corresponding ligand to initiate immunosuppressive effects on T cells. As a result, the function of immune cells infiltrating the tumor’s vicinity is diminished, enabling tumor cells to evade surveillance and clearance by the body.^[10]^

PD-1 inhibitors disrupt the PD-1/PD-L1 pathway, which then triggers autoimmune responses and directly activates CD8 + T lymphocytes. Once activated, these T cells effectively infiltrate and eliminate tumor cells.^[11]^ However, in this process they also infiltrate and destroy islet cells, accelerating the deterioration of islet function and resulting in insulin deficiency. Crucially, this impairment of islet function can be irreversible and does not tend to recover spontaneously.

In this case, when high blood glucose was initially observed, the patient’s serum C-peptide level was 0.34 ng/mL. However, after 10 days, the serum C-peptide dropped to <0.01 ng/mL and insulin levels were <0.20 μmol/L, suggesting potential irreversible damage to the patient’s islet cells. The mechanisms leading to lactic acidosis during hyperglycemia in this case can be explained as follows: First, ICIs caused an acute impairment of islet function, resulting in an absolute insulin deficiency in the body. As a result, the decreased insulin/glucagon ratio increased gluconeogenesis, glycogenolysis and hepatic ketogenesis. In addition, patients with bronchiectasis in both lungs may experience tissue hypoxia, leading to mitochondrial dysfunction in the affected cells and conversion of pyruvate to lactate in the cytoplasm. Secondly, cellular dehydration and osmotic diuresis during hyperglycemia, accompanied by nausea and vomiting during the compensatory phase of acidosis, contribute to water loss in the body. This leads to inadequate circulating blood volume, relative hypoxia in various tissues and organs, and reduced oxidative utilization of lactic acid or conversion to glycogen pathways in the liver. Thirdly, reduced renal perfusion due to insufficient circulating blood volume leads to reduced renal excretion of lactic acid, further contributing to and exacerbating lactic acidosis.

Regarding the transient hyperuricemia observed in the patient on both occasions, it is suggested by the authors that the high glucose environment may have increased uric acid production by facilitating the breakdown of adenosine triphosphate to adenosine, which is subsequently converted to xanthine. Reduced renal perfusion also contributes to reduced uric acid excretion. Although fewer cases of ICI-DM resulting in lactic acidosis and hyperuricemia have been reported, further prospective studies are necessary to fully elucidate the precise underlying mechanisms involved.

Skin toxicity is a common irAEs,^[12]^ and the majority of these cutaneous adverse events are self-limiting and can be alleviated with conventional treatment. The exact mechanism of cutaneous adverse events remains unknown, but it may be associated with ICIs interfering with the activation of T-cells mediated by checkpoint receptors.^[13]^ The PD-1 checkpoint acts as a regulatory factor in the immune system, dampening the activity of T-cells. However, ICIs obstruct the PD-1/PD-L1 pathway, leading to excessive T-cell activation and upregulation of Th17 cell activity, resulting in the production of significant amounts of interleukins. This ultimately triggers rapid epidermal turnover. This immune mechanism has similarities to psoriasis, where antigen-presenting cells produce IL-23, which induces the proliferation and differentiation of Th17 cells and the production of various Th17-like cytokines. This results in excessive proliferation of keratinocytes.^[14]^ In this case, the patient had previously had mild symptoms of psoriasis and this episode was considered a recurrence.

In the treatment of psoriasis, topical glucocorticoids, narrow-spectrum ultraviolet irradiation, and oral anti-silver granules have provided significant relief. Systemic glucocorticoid or anti-TNF-α therapy may be used appropriately in severe cases, although the focus should be on the individual systemic disease. One case reported that the use of interleukin-17A (IL-17A) inhibitor (Secukinumab) can effectively control patients with critical illness.^[15]^ In the management of ICI-DM, the impairment of islet function by ICIs is irreversible, so patients can only be treated with insulin hypoglycemia. When deciding whether to discontinue or reactivate ICIs therapy, the US guidelines^[16]^ suggest that CTCAE classification can be performed according to the patient’s fasting glucose: <160 mg/dL (8.9 mmol/L) is grade 1, 160–250 mg/dL (8.9–13.9 mmol/L) is grade 2, 250–500 mg/dL (13.9–27.8 mmol/L) is grade 3, and >500 mg/dL (27.8 mmol/L) is grade 4. Patients with CTCAE ≤ grade 1 may continue ICIs therapy while initiating insulin for glycemic control; patients with CTCAE ≥ grade 3 should discontinue ICIs therapy, and ICIs may be restarted when glycemia returns to CTCAE ≤ grade 1. In this case, the patient’s fasting glucose was mostly stable at CTCAE grade 2, so ICIs were not restarted after discontinuation.

## 4. Conclusions

In summary, the use of ICIs in patients with tumor-associated autoimmune diseases inevitably leads to a variety of complex irAEs manifestations. Most clinical trials of ICIs exclude patients with concomitant autoimmune diseases to avoid exacerbation of preexisting autoimmune diseases or increased risk of irAEs after ICI administration.^[17]^ Researchers believe that the incidence of irAEs is higher in patients with a history of autoimmune disease or positive auto insulin antibodies prior to receiving ICIs. The safety and efficacy of ICIs in patients with autoimmune diseases are still not well established, emphasizing the need to improve clinicians’ early recognition of these conditions. It has been suggested^[18]^ that certain biomarkers, such as carcinoembryonic antigen-associated cell adhesion factor-1 (CECAM1) and CD177, may serve as predictors of ICIs-induced endocrine adverse effects. These biomarkers may help clinicians to identify patients with ICI-DM at an early stage. Future research can focus on the early detection of effective biomarkers of irAEs, exploring ways to reverse or delay organ damage, and investigating the relationship between preexisting autoimmune diseases and the efficacy of antitumor immunotherapy.

## Acknowledgments

We extend our gratitude to our patient for her support.

## Author contributions

**Conceptualization:** Min Li,Wenying Huang.

**Data curation:** Yuan Xue,Wenying Huang.

**Formal analysis:** Wenying Huang.

**Funding acquisition:** Ying Guo,Wenying Huang.

**Investigation:** Min Li, Wenying Huang.

**Methodology:** Yan Liu, Wenying Huang.

**Project administration:** Ying Guo, Wenying Huang.

**Resources:** Ying Guo,Wenying Huang, Yan Liu.

**Software:** Yan Liu, Wenying Huang.

**Supervision:** Wenying Huang, Ying Guo.

**Validation:** Wenying Huang.

**Visualization:** Weichao Bao, Wenying Huang.

**Writing – original draft:** Wenying Huang, Yuan Xue.

**Writing – review & editing:** Wenying Huang, Weichao Bao.
